# 1-Benzoyl-3-methyl-2,6-diphenyl-4-piperidone

**DOI:** 10.1107/S1600536809023848

**Published:** 2009-06-27

**Authors:** P. Nithya, Venkatesha R. Hathwar, Sriramakrishnaswamy Kone, N. Malathi, F. Nawaz Khan

**Affiliations:** a Chemistry Division, School of Science and Humanities, VIT University, Vellore 632 014, Tamil Nadu, India; b Solid State and Structural Chemistry Unit, Indian Institute of Science, Bangalore 560 012, Karnataka, India

## Abstract

In the title moleclue, C_25_H_23_NO_2_, the 4-piperidone ring adopts a boat conformation. The mol­ecular conformation is stabilized by an intra­molecular C—H⋯O hydrogen bond. In the crystal, mol­ecules are connected through weak inter­molecular C—H⋯O hydrogen bonds.

## Related literature

For general background, see: Grishina *et al.* (1994 ▶); Nalanishi *et al.* (1974 ▶); Perumal *et al.* (2001 ▶); Ponnuswamy *et al.* (2002 ▶). For related structures, see: Gayathri *et al.* (2008 ▶); Nithya *et al.* (2009 ▶). For details of the synthesis, see: Noller & Baliah (1948 ▶).
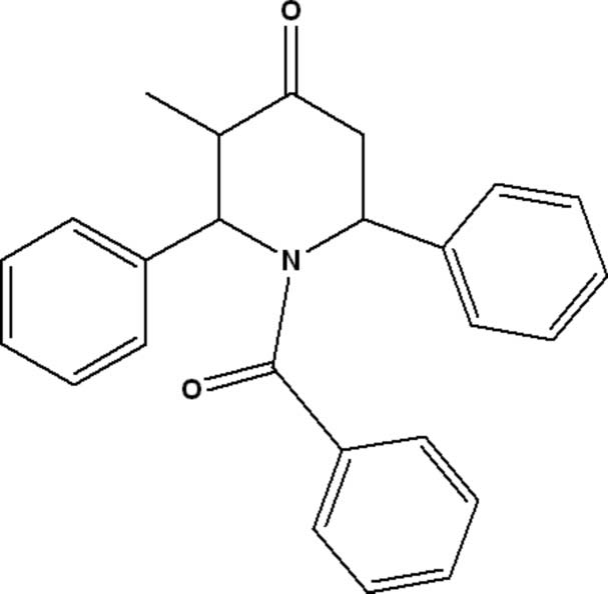


## Experimental

### 

#### Crystal data

C_25_H_23_NO_2_
*M* *_r_* = 369.44
Monoclinic, 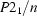

*a* = 11.7602 (6) Å
*b* = 9.2404 (3) Å
*c* = 19.1722 (9) Å
β = 98.797 (4)°
*V* = 2058.93 (16) Å^3^
*Z* = 4
Mo *K*α radiation
μ = 0.08 mm^−1^
*T* = 290 K
0.36 × 0.24 × 0.18 mm

#### Data collection

Oxford Xcalibur Eos(Nova) CCD detector diffractometer
Absorption correction: multi-scan (*CrysAlis RED*; Oxford Diffraction, 2009 ▶) *T*_min_ = 0.942, *T*_max_ = 0.987
22931 measured reflections
3831 independent reflections
2515 reflections with *I* > 2σ(*I*)
*R*_int_ = 0.044

#### Refinement

*R*[*F* ^2^ > 2σ(*F* ^2^)] = 0.042
*wR*(*F* ^2^) = 0.105
*S* = 1.00
3831 reflections
254 parameters
H-atom parameters constrained
Δρ_max_ = 0.12 e Å^−3^
Δρ_min_ = −0.15 e Å^−3^

### 

Data collection: *CrysAlis CCD* (Oxford Diffraction, 2009 ▶); cell refinement: *CrysAlis CCD*; data reduction: *CrysAlis RED* (Oxford Diffraction, 2009 ▶); program(s) used to solve structure: *SHELXS97* (Sheldrick, 2008 ▶); program(s) used to refine structure: *SHELXL97* (Sheldrick, 2008 ▶); molecular graphics: *ORTEP-3* (Farrugia, 1997 ▶); software used to prepare material for publication: *WinGX* (Farrugia, 1999 ▶).

## Supplementary Material

Crystal structure: contains datablocks global, I. DOI: 10.1107/S1600536809023848/bt2975sup1.cif

Structure factors: contains datablocks I. DOI: 10.1107/S1600536809023848/bt2975Isup2.hkl

Additional supplementary materials:  crystallographic information; 3D view; checkCIF report

## Figures and Tables

**Table 1:** Hydrogen-bond geometry (Å, °)

*D*—H⋯*A*	*D*—H	H⋯*A*	*D*⋯*A*	*D*—H⋯*A*
C8—H8⋯O1	0.98	2.26	2.7235 (17)	108
C9—H9*A*⋯O1^i^	0.97	2.56	3.4446 (19)	152

Symmetry code: (i) 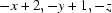.

**Fig. 1.**
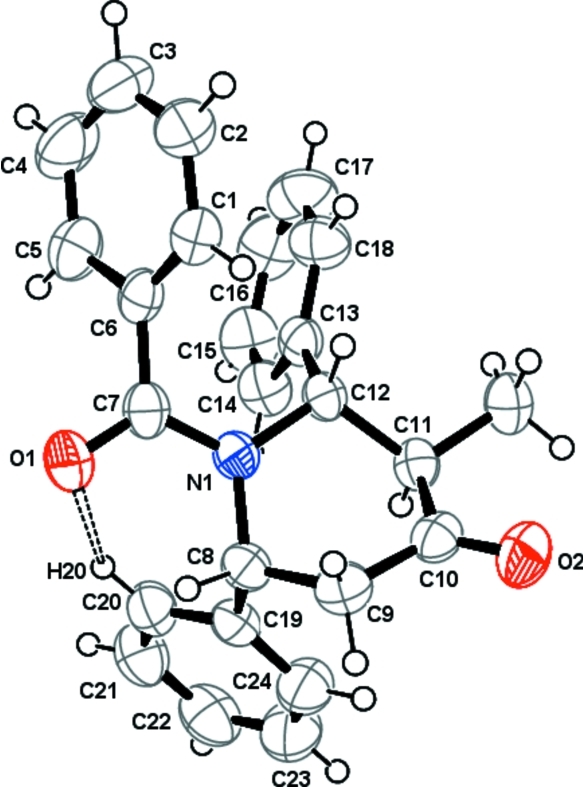
ORTEP diagram of the title compound with 50% probability displacement ellipsoids. Dotted bond indicates the intramolecular C—H···O hydrogen bond.

**Fig. 2.**
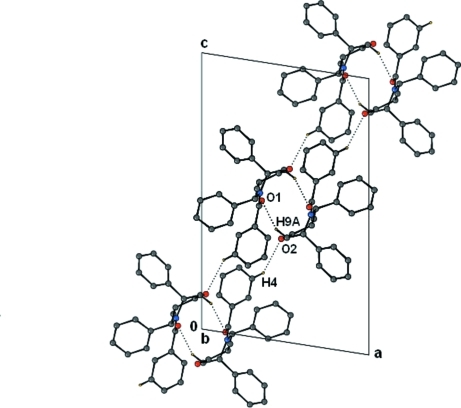
Crystal packing diagram of the title compound. The dotted lines indicate intermolecular C—H···O hydrogen bonds.

## supplementary crystallographic information

### Comment

4-piperidones and their derivatives present potential medical applications (Grishina *et al.*, 1994, Ponnuswamy *et al.*, 2002, Nalanishi *et al.*, 1974). Piperidones are also reported to possess analgesic,anti-inflammatory, central nervous system (*CNS*), local anaesthetic, anticancer and antimicrobial activity (Perumal *et al.*, 2001). In continouus of our interest in piperidones (Nithya *et al.*, 2009), the crystal structure of title compound is discussed in this paper.

In the title molecule, C_25_H_23_NO_2_ (Fig. 1), the piperidine ring adopts a boat conformation.   In the related crystal structure, the piperidine ring also adopts a chair conformation (Gayathri *et al.*, 2008) but the three substituents on the C atoms of the ring are in axial orientations. In the crystal, the molecules are connected through weak intermolecular C—H···O hydrogen bonds. (Fig. 2).

### Experimental

To a well stirred solution of 3 methyl-2,6-bis(phenyl)piperidin-4-one (1 equiv.) and triethylamine (1 equiv.) in freshly distilled benzene, benzoyl chloride (1 equiv.) in benzene was added dropwise. Stirring was continued until the completion of reaction. Later, it was poured into water and extracted with DCM, washed well with sodium bicarbonate solution and dried over anhydrous sodium sulfate. This upon evaporation and subsequent recrystallization in distilled ethanol furnished the diffraction-quality crystals of the title compound.

### Refinement

All H atoms in were positioned geometrically and refined using a riding model with C—H bond lenghts of 0.93, 0.97 and 0.96Å for aromatic, methylene and methyl H atoms respectively and *U*_iso_(H) = 1.2*U*_eq_(C) or 1.5*U*_eq_(C_methyl_).

### Crystal data

**Table:**

C_25_H_23_NO_2_	*F*(000) = 784
*M**_r_* = 369.44	*D*_x_ = 1.192 Mg m^−^^3^
Monoclinic, *P*2_1_/*n*	Mo *K*α radiation, λ = 0.71073 Å
Hall symbol: -P 2yn	Cell parameters from 983 reflections
*a* = 11.7602 (6) Å	θ = 2.0–21.3°
*b* = 9.2404 (3) Å	µ = 0.08 mm^−^^1^
*c* = 19.1722 (9) Å	*T* = 290 K
β = 98.797 (4)°	Block, colorless
*V* = 2058.93 (16) Å^3^	0.36 × 0.24 × 0.18 mm
*Z* = 4	

### Data collection

**Table:**

Oxford Xcalibur Eos(Nova) CCD detector diffractometer	3831 independent reflections
Radiation source: Enhance (Mo) X-ray Source	2515 reflections with *I* > 2σ(*I*)
graphite	*R*_int_ = 0.044
ω scans	θ_max_ = 25.5°, θ_min_ = 2.9°
Absorption correction: multi-scan (*CrysAlis RED*; Oxford Diffraction, 2009)	*h* = −14→14
*T*_min_ = 0.942, *T*_max_ = 0.987	*k* = −11→11
22931 measured reflections	*l* = −23→23

### Refinement

**Table:**

Refinement on *F*^2^	Primary atom site location: structure-invariant direct methods
Least-squares matrix: full	Secondary atom site location: difference Fourier map
*R*[*F*^2^ > 2σ(*F*^2^)] = 0.042	Hydrogen site location: inferred from neighbouring sites
*wR*(*F*^2^) = 0.105	H-atom parameters constrained
*S* = 1.00	*w* = 1/[σ^2^(*F*_o_^2^) + (0.0536*P*)^2^] where *P* = (*F*_o_^2^ + 2*F*_c_^2^)/3
3831 reflections	(Δ/σ)_max_ < 0.001
254 parameters	Δρ_max_ = 0.12 e Å^−^^3^
0 restraints	Δρ_min_ = −0.15 e Å^−^^3^

### Special details

**Table:**

Geometry. All e.s.d.'s (except the e.s.d. in the dihedral angle between two l.s. planes) are estimated using the full covariance matrix. The cell e.s.d.'s are taken into account individually in the estimation of e.s.d.'s in distances, angles and torsion angles; correlations between e.s.d.'s in cell parameters are only used when they are defined by crystal symmetry. An approximate (isotropic) treatment of cell e.s.d.'s is used for estimating e.s.d.'s involving l.s. planes.
Refinement. Refinement of *F*^2^ against ALL reflections. The weighted *R*-factor *wR* and goodness of fit *S* are based on *F*^2^, conventional *R*-factors *R* are based on *F*, with *F* set to zero for negative *F*^2^. The threshold expression of *F*^2^ > σ(*F*^2^) is used only for calculating *R*-factors(gt) *etc*. and is not relevant to the choice of reflections for refinement. *R*-factors based on *F*^2^ are statistically about twice as large as those based on *F*, and *R*- factors based on ALL data will be even larger.

### Fractional atomic coordinates and isotropic or equivalent isotropic displacement parameters (Å^2^)

**Table:**

	*x*	*y*	*z*	*U*_iso_*/*U*_eq_	
N1	0.84703 (9)	0.33889 (11)	0.02444 (6)	0.0410 (3)	
O1	0.85789 (10)	0.57741 (11)	−0.00176 (6)	0.0660 (3)	
O2	1.02467 (10)	0.01000 (12)	0.12851 (6)	0.0696 (4)	
C1	0.89524 (13)	0.34406 (15)	−0.13261 (8)	0.0502 (4)	
H1	0.9616	0.3060	−0.1064	0.060*	
C2	0.87411 (16)	0.32413 (18)	−0.20472 (9)	0.0660 (5)	
H2	0.9268	0.2739	−0.2271	0.079*	
C3	0.77585 (19)	0.3780 (2)	−0.24350 (10)	0.0777 (6)	
H3	0.7610	0.3623	−0.2920	0.093*	
C4	0.69948 (17)	0.4549 (2)	−0.21091 (11)	0.0813 (6)	
H4	0.6327	0.4913	−0.2373	0.098*	
C5	0.72094 (15)	0.47875 (17)	−0.13942 (10)	0.0644 (5)	
H5	0.6701	0.5342	−0.1179	0.077*	
C6	0.81811 (12)	0.42043 (14)	−0.09919 (8)	0.0444 (4)	
C7	0.84216 (12)	0.45127 (15)	−0.02206 (8)	0.0458 (4)	
C8	0.89332 (12)	0.36834 (15)	0.09936 (7)	0.0446 (4)	
H8	0.9286	0.4645	0.1003	0.053*	
C9	0.99148 (12)	0.26268 (15)	0.12180 (8)	0.0509 (4)	
H9A	1.0553	0.2873	0.0974	0.061*	
H9B	1.0175	0.2739	0.1720	0.061*	
C10	0.95985 (13)	0.10771 (16)	0.10705 (8)	0.0460 (4)	
C11	0.84348 (12)	0.07997 (14)	0.06341 (7)	0.0423 (4)	
H11	0.7854	0.0968	0.0941	0.051*	
C12	0.81844 (11)	0.18755 (14)	0.00098 (7)	0.0397 (3)	
H12	0.8685	0.1616	−0.0335	0.048*	
C13	0.69509 (12)	0.17244 (14)	−0.03516 (8)	0.0437 (4)	
C14	0.60407 (14)	0.21590 (17)	−0.00264 (9)	0.0575 (4)	
H14	0.6182	0.2590	0.0417	0.069*	
C15	0.49176 (15)	0.1960 (2)	−0.03526 (11)	0.0741 (5)	
H15	0.4312	0.2270	−0.0130	0.089*	
C16	0.46924 (17)	0.1311 (2)	−0.09994 (12)	0.0814 (6)	
H16	0.3937	0.1163	−0.1213	0.098*	
C17	0.55856 (18)	0.0882 (2)	−0.13305 (10)	0.0820 (6)	
H17	0.5437	0.0454	−0.1774	0.098*	
C18	0.67089 (15)	0.10815 (17)	−0.10088 (9)	0.0627 (5)	
H18	0.7310	0.0779	−0.1238	0.075*	
C19	0.80010 (13)	0.37833 (15)	0.14586 (8)	0.0467 (4)	
C20	0.71183 (14)	0.47793 (16)	0.12868 (9)	0.0580 (4)	
H20	0.7110	0.5362	0.0891	0.070*	
C21	0.62531 (16)	0.49192 (18)	0.16932 (10)	0.0708 (5)	
H21	0.5665	0.5585	0.1568	0.085*	
C22	0.62607 (17)	0.4075 (2)	0.22834 (11)	0.0751 (5)	
H22	0.5681	0.4172	0.2560	0.090*	
C23	0.71230 (17)	0.3091 (2)	0.24616 (10)	0.0790 (6)	
H23	0.7127	0.2512	0.2859	0.095*	
C24	0.79907 (15)	0.29535 (18)	0.20530 (9)	0.0651 (5)	
H24	0.8578	0.2287	0.2183	0.078*	
C25	0.83049 (14)	−0.07659 (16)	0.03829 (9)	0.0588 (4)	
H25A	0.8866	−0.0972	0.0082	0.088*	
H25B	0.7547	−0.0909	0.0126	0.088*	
H25C	0.8421	−0.1402	0.0783	0.088*	

### Atomic displacement parameters (Å^2^)

**Table:**

	*U*^11^	*U*^22^	*U*^33^	*U*^12^	*U*^13^	*U*^23^
N1	0.0435 (7)	0.0352 (6)	0.0436 (7)	−0.0058 (5)	0.0047 (6)	−0.0012 (5)
O1	0.0943 (9)	0.0367 (6)	0.0664 (8)	−0.0083 (6)	0.0106 (6)	0.0005 (5)
O2	0.0658 (8)	0.0648 (7)	0.0731 (8)	0.0187 (6)	−0.0059 (6)	0.0089 (6)
C1	0.0486 (10)	0.0461 (8)	0.0549 (10)	0.0004 (7)	0.0054 (8)	0.0059 (7)
C2	0.0783 (13)	0.0635 (11)	0.0584 (12)	−0.0004 (9)	0.0178 (10)	0.0029 (9)
C3	0.0997 (16)	0.0817 (13)	0.0488 (11)	−0.0026 (12)	0.0016 (11)	0.0111 (10)
C4	0.0782 (14)	0.0880 (14)	0.0684 (14)	0.0109 (11)	−0.0188 (11)	0.0197 (11)
C5	0.0589 (11)	0.0628 (10)	0.0694 (13)	0.0128 (8)	0.0032 (9)	0.0124 (9)
C6	0.0434 (9)	0.0375 (7)	0.0514 (10)	−0.0046 (7)	0.0042 (7)	0.0080 (7)
C7	0.0429 (9)	0.0381 (8)	0.0570 (10)	−0.0028 (6)	0.0096 (7)	0.0036 (7)
C8	0.0457 (9)	0.0403 (8)	0.0462 (9)	−0.0068 (6)	0.0025 (7)	−0.0035 (6)
C9	0.0448 (9)	0.0571 (9)	0.0501 (10)	−0.0043 (7)	0.0051 (7)	−0.0005 (7)
C10	0.0459 (9)	0.0530 (9)	0.0412 (9)	0.0058 (7)	0.0132 (7)	0.0062 (7)
C11	0.0409 (8)	0.0381 (7)	0.0499 (9)	−0.0006 (6)	0.0134 (7)	0.0033 (6)
C12	0.0402 (8)	0.0341 (7)	0.0455 (8)	−0.0030 (6)	0.0082 (6)	−0.0006 (6)
C13	0.0448 (9)	0.0357 (7)	0.0495 (9)	−0.0065 (6)	0.0039 (7)	0.0023 (6)
C14	0.0470 (10)	0.0558 (9)	0.0681 (11)	−0.0046 (8)	0.0037 (9)	−0.0043 (8)
C15	0.0456 (11)	0.0762 (12)	0.0989 (15)	−0.0021 (9)	0.0056 (10)	−0.0002 (11)
C16	0.0551 (13)	0.0854 (14)	0.0945 (16)	−0.0163 (10)	−0.0178 (11)	0.0089 (12)
C17	0.0756 (14)	0.0943 (14)	0.0688 (13)	−0.0248 (12)	−0.0122 (11)	−0.0089 (11)
C18	0.0601 (11)	0.0669 (11)	0.0595 (11)	−0.0134 (9)	0.0036 (9)	−0.0088 (9)
C19	0.0495 (9)	0.0436 (8)	0.0463 (9)	−0.0037 (7)	0.0048 (7)	−0.0075 (7)
C20	0.0656 (12)	0.0479 (9)	0.0615 (11)	0.0058 (8)	0.0131 (9)	−0.0020 (8)
C21	0.0699 (13)	0.0615 (11)	0.0843 (14)	0.0134 (9)	0.0221 (11)	−0.0084 (10)
C22	0.0775 (14)	0.0742 (12)	0.0812 (14)	−0.0013 (11)	0.0370 (11)	−0.0141 (11)
C23	0.0956 (15)	0.0831 (13)	0.0641 (12)	0.0116 (12)	0.0309 (11)	0.0071 (10)
C24	0.0705 (12)	0.0706 (11)	0.0567 (11)	0.0142 (9)	0.0179 (9)	0.0043 (9)
C25	0.0631 (11)	0.0419 (9)	0.0715 (12)	−0.0009 (7)	0.0102 (9)	0.0042 (8)

### Geometric parameters (Å, °)

**Table:**

N1—C7	1.3641 (17)	C12—C13	1.5154 (19)
N1—C8	1.4813 (17)	C12—H12	0.9800
N1—C12	1.4916 (16)	C13—C14	1.378 (2)
O1—C7	1.2338 (16)	C13—C18	1.383 (2)
O2—C10	1.2123 (16)	C14—C15	1.385 (2)
C1—C2	1.379 (2)	C14—H14	0.9300
C1—C6	1.3818 (19)	C15—C16	1.366 (3)
C1—H1	0.9300	C15—H15	0.9300
C2—C3	1.369 (2)	C16—C17	1.367 (3)
C2—H2	0.9300	C16—H16	0.9300
C3—C4	1.369 (3)	C17—C18	1.382 (2)
C3—H3	0.9300	C17—H17	0.9300
C4—C5	1.373 (2)	C18—H18	0.9300
C4—H4	0.9300	C19—C24	1.375 (2)
C5—C6	1.386 (2)	C19—C20	1.389 (2)
C5—H5	0.9300	C20—C21	1.379 (2)
C6—C7	1.490 (2)	C20—H20	0.9300
C8—C19	1.5179 (19)	C21—C22	1.374 (2)
C8—C9	1.523 (2)	C21—H21	0.9300
C8—H8	0.9800	C22—C23	1.366 (3)
C9—C10	1.496 (2)	C22—H22	0.9300
C9—H9A	0.9700	C23—C24	1.384 (2)
C9—H9B	0.9700	C23—H23	0.9300
C10—C11	1.513 (2)	C24—H24	0.9300
C11—C25	1.525 (2)	C25—H25A	0.9600
C11—C12	1.5491 (19)	C25—H25B	0.9600
C11—H11	0.9800	C25—H25C	0.9600
			
C7—N1—C8	117.80 (11)	C13—C12—C11	110.48 (10)
C7—N1—C12	122.12 (11)	N1—C12—H12	107.5
C8—N1—C12	119.80 (10)	C13—C12—H12	107.5
C2—C1—C6	120.18 (15)	C11—C12—H12	107.5
C2—C1—H1	119.9	C14—C13—C18	118.13 (14)
C6—C1—H1	119.9	C14—C13—C12	121.44 (13)
C3—C2—C1	120.25 (17)	C18—C13—C12	120.37 (13)
C3—C2—H2	119.9	C13—C14—C15	120.67 (16)
C1—C2—H2	119.9	C13—C14—H14	119.7
C2—C3—C4	119.96 (18)	C15—C14—H14	119.7
C2—C3—H3	120.0	C16—C15—C14	120.49 (17)
C4—C3—H3	120.0	C16—C15—H15	119.8
C3—C4—C5	120.37 (17)	C14—C15—H15	119.8
C3—C4—H4	119.8	C15—C16—C17	119.53 (17)
C5—C4—H4	119.8	C15—C16—H16	120.2
C4—C5—C6	120.21 (17)	C17—C16—H16	120.2
C4—C5—H5	119.9	C16—C17—C18	120.27 (18)
C6—C5—H5	119.9	C16—C17—H17	119.9
C1—C6—C5	118.97 (15)	C18—C17—H17	119.9
C1—C6—C7	121.30 (13)	C17—C18—C13	120.90 (17)
C5—C6—C7	119.54 (14)	C17—C18—H18	119.6
O1—C7—N1	121.58 (14)	C13—C18—H18	119.6
O1—C7—C6	119.37 (12)	C24—C19—C20	117.71 (14)
N1—C7—C6	119.05 (12)	C24—C19—C8	123.47 (14)
N1—C8—C19	112.91 (11)	C20—C19—C8	118.81 (13)
N1—C8—C9	107.85 (11)	C21—C20—C19	121.17 (16)
C19—C8—C9	117.22 (12)	C21—C20—H20	119.4
N1—C8—H8	106.0	C19—C20—H20	119.4
C19—C8—H8	106.0	C22—C21—C20	119.99 (17)
C9—C8—H8	106.0	C22—C21—H21	120.0
C10—C9—C8	113.86 (12)	C20—C21—H21	120.0
C10—C9—H9A	108.8	C23—C22—C21	119.69 (17)
C8—C9—H9A	108.8	C23—C22—H22	120.2
C10—C9—H9B	108.8	C21—C22—H22	120.2
C8—C9—H9B	108.8	C22—C23—C24	120.21 (17)
H9A—C9—H9B	107.7	C22—C23—H23	119.9
O2—C10—C9	121.58 (14)	C24—C23—H23	119.9
O2—C10—C11	122.02 (13)	C19—C24—C23	121.23 (16)
C9—C10—C11	116.40 (12)	C19—C24—H24	119.4
C10—C11—C25	111.95 (12)	C23—C24—H24	119.4
C10—C11—C12	111.57 (11)	C11—C25—H25A	109.5
C25—C11—C12	111.51 (12)	C11—C25—H25B	109.5
C10—C11—H11	107.2	H25A—C25—H25B	109.5
C25—C11—H11	107.2	C11—C25—H25C	109.5
C12—C11—H11	107.2	H25A—C25—H25C	109.5
N1—C12—C13	112.34 (11)	H25B—C25—H25C	109.5
N1—C12—C11	111.18 (11)		
			
C6—C1—C2—C3	−1.0 (2)	C7—N1—C12—C11	173.99 (12)
C1—C2—C3—C4	1.6 (3)	C8—N1—C12—C11	0.20 (16)
C2—C3—C4—C5	0.1 (3)	C10—C11—C12—N1	−46.08 (15)
C3—C4—C5—C6	−2.4 (3)	C25—C11—C12—N1	−172.09 (11)
C2—C1—C6—C5	−1.3 (2)	C10—C11—C12—C13	−171.51 (11)
C2—C1—C6—C7	−176.24 (13)	C25—C11—C12—C13	62.48 (15)
C4—C5—C6—C1	3.0 (2)	N1—C12—C13—C14	−54.97 (17)
C4—C5—C6—C7	178.01 (15)	C11—C12—C13—C14	69.80 (16)
C8—N1—C7—O1	−11.4 (2)	N1—C12—C13—C18	127.97 (14)
C12—N1—C7—O1	174.69 (12)	C11—C12—C13—C18	−107.26 (15)
C8—N1—C7—C6	168.16 (12)	C18—C13—C14—C15	−0.3 (2)
C12—N1—C7—C6	−5.76 (19)	C12—C13—C14—C15	−177.39 (14)
C1—C6—C7—O1	113.48 (16)	C13—C14—C15—C16	0.9 (3)
C5—C6—C7—O1	−61.43 (19)	C14—C15—C16—C17	−1.2 (3)
C1—C6—C7—N1	−66.08 (18)	C15—C16—C17—C18	1.0 (3)
C5—C6—C7—N1	119.00 (16)	C16—C17—C18—C13	−0.4 (3)
C7—N1—C8—C19	103.78 (14)	C14—C13—C18—C17	0.1 (2)
C12—N1—C8—C19	−82.15 (15)	C12—C13—C18—C17	177.22 (15)
C7—N1—C8—C9	−125.09 (13)	N1—C8—C19—C24	124.75 (15)
C12—N1—C8—C9	48.98 (15)	C9—C8—C19—C24	−1.5 (2)
N1—C8—C9—C10	−52.73 (15)	N1—C8—C19—C20	−56.42 (17)
C19—C8—C9—C10	76.00 (16)	C9—C8—C19—C20	177.30 (13)
C8—C9—C10—O2	−172.09 (13)	C24—C19—C20—C21	−0.8 (2)
C8—C9—C10—C11	8.52 (17)	C8—C19—C20—C21	−179.69 (14)
O2—C10—C11—C25	−11.66 (19)	C19—C20—C21—C22	0.6 (3)
C9—C10—C11—C25	167.74 (12)	C20—C21—C22—C23	−0.5 (3)
O2—C10—C11—C12	−137.43 (14)	C21—C22—C23—C24	0.5 (3)
C9—C10—C11—C12	41.97 (16)	C20—C19—C24—C23	0.8 (2)
C7—N1—C12—C13	−61.62 (16)	C8—C19—C24—C23	179.67 (15)
C8—N1—C12—C13	124.58 (13)	C22—C23—C24—C19	−0.7 (3)

### Hydrogen-bond geometry (Å, °)

**Table:**

*D*—H···*A*	*D*—H	H···*A*	*D*···*A*	*D*—H···*A*
C8—H8···O1	0.98	2.26	2.7235 (17)	108
C9—H9A···O1^i^	0.97	2.56	3.4446 (19)	152

Symmetry codes: (i) −*x*+2, −*y*+1, −*z*.
